# Evidence for proactive and reactive helping in two- to five-year-olds from a small-scale society

**DOI:** 10.1371/journal.pone.0187787

**Published:** 2017-11-15

**Authors:** Hilary Aime, Tanya Broesch, Lara B. Aknin, Felix Warneken

**Affiliations:** 1 Department of Psychology, Simon Fraser University, Burnaby, British Columbia, Canada; 2 Department of Psychology, University of Michigan, Ann Arbor, Michigan, United States of America; Leipzig University, GERMANY

## Abstract

Humans are unique in their propensity for helping. Not only do we help others in need by *reacting* to their requests, we also help *proactively* by assisting in the absence of a request. Proactive helping requires the actor to detect the need for help, recognize the intention of the other, and remedy the situation. Very little is known about the development of this social phenomenon beyond an urban, industrialized setting. We examined helping in nineteen two- to five-year old children in small-scale rural villages of Vanuatu. In the experimental condition, the intentions of the experimenter were made salient, whereas in the control condition they were ambiguous. Children helped more often in the experimental compared to the control condition, suggesting that the propensity to monitor others’ goals and act accordingly can be detected in different cultural contexts.

## Introduction

Helpfulness in early childhood is interpreted as early evidence of cooperation–a central aspect of human social life [1–3]. The most frequently studied form of prosocial behavior involves reactive helping–providing assistance in response to a clear behavioral cue or direct communicative request for help [4]. Research suggests that this ability is present in young children across diverse societies from around the world [5–7]. However, recent work also demonstrates that young children from North America can help *proactively* by offering assistance in the absence of a clear request for help, namely in situations in which the target needing help is not even aware that a problem exists, such as an object falling down while she is turned away [4]. This gives insight into children’s cognitive abilities because proactive helping requires that children are able to infer a person’s need for help from the situation without relying on behavioral or communicative cues. Moreover, proactive helping is spontaneous, with children offering help in the absence of any direct or indirect requests. Is the proclivity to engage in proactive helping present among children from cultures outside North America? Past work has not addressed this question and has instead extrapolated from Western samples to humans more generally. Here, we investigate whether young children between the ages of 2 to 5 years from a rural, non-Western society in Vanuatu offer assistance in the absence of a clear cue for help.

### Reactive helping

People frequently provide information to signal when they are in need of assistance so helpers can respond appropriately. Reactive helping requires the helper to both recognize and respond to overt behavioral cues from the helpee. Past research conducted in Western cultures, such as North America and Europe, past research has found that children not only provide assistance to others when needed [1–3, 8], but that children pay attention to a variety of cues that may signal help is required [2, 9–11]. Indeed, much of the research on helping in children relies on overt behavioral cues provided by the individual in need of help and, perhaps not surprisingly, finds that children are more likely to respond when cues are direct. For example, past research has shown that children as young as 18 months of age provide assistance when a helpee reaches for the fallen object [2], makes a negative facial expression [9, 10], or verbally states their desire for assistance [11]. Helping also becomes more likely as the behavioral and communicative cues soliciting the help become more explicit [10].

Furthermore, research indicates that young children across diverse societies will provide assistance in response to a behavioural cue. The propensity for children to offer reactive help during 18- to 24-months of age has been examined in three cross-cultural explorations with evidence from young children in Peru, India, and both urban and rural Canada [5]; both urban and rural Brazil and Germany [6]; and India and Germany [7]. In all three studies, children recognized and responded to an overt cue or request for help within the second year of life. Interestingly, the researchers reported some cultural differences in the rates of reactive helping [7], as well as differences in the ways in which helping is socialized [6]. For instance, Ginner Torréns and Kärtner reported children in India helped more than their German counterparts, and mothers in India used more punitive practices when children did not follow a helping request and praised their children less for helping [7]. Taken together, this work demonstrates consistency in the proclivity for children across cultures to engage in helping, but variation in the frequency and motivations for the behavior.

Despite the evidence demonstrating that children become increasingly proficient at helping others in the second year of life, how infants develop this proficiency has yet to established. Understanding the nature of how children’s helping develops in important for our interpretation of cross-cultural findings regarding similarities and differences in children’s helping. One explanation of prosocial behaviour early in development is that children have a natural tendency to help others that emerges with little influence from early experience [12, 13]. This position is supported by evidence that documents commonalities across cultures in the occurrence of helping behaviors [5–7]. Other perspectives indicate that early helping develops through experiences in everyday social interactions [14–17]. From this perspective, children have social experiences that contribute to the emergence and development of helping. For example, children’s experience of requests, values of obedience, praise, and punishment during their participation in daily activities with family members facilitates and guides their helping behavior [15]. In line with this perspective, research by Dahl and colleagues shows that young children’s helping behavior increased both during and after explicit scaffolding from an adult, underscoring the importance of socialization in the development of prosocial behavior [16].

### Proactive helping

A key component of human prosociality is that people assist one another even when help is unsolicited, including situations in which the target is unaware of a problem and thus cannot indicate that intervention is necessary [4, 8, 18]. For example, a helper may run after a patron at a coffee shop who unknowingly left his or her jacket on a chair, or return a wallet to the pedestrian who accidentally dropped it while crossing the street. These demonstrations of *proactive helping* are particularly complex because they require the helper to identify the intentions or desires of the individual as well as the incongruence of the situation with those desires (for alternative perspectives on the interpretation of children’s motivation and behavior, see [19, 20]). Indeed, proactive helping behavior requires a more complex understanding of other’s intentions and desires than reactive helping, as the helping occurs in the absence of any behavioral or communicative cues indicating a need for help, as well as in the absence of any behavior that could be interpreted as a communicative request.

Despite these challenges, the propensity to engage in proactive helping emerges in North American children during toddlerhood. In the first demonstration of this effect [4], Warneken exposed young children to the desires of an experimenter who wanted a fallen object (a can) to either remain on the ground or be picked up. Then, moments later, the experimenter dropped the same object without noticing. By 25 months of age, children responded by picking up the object *only* when doing so matched the experimenter’s desires, as demonstrated by their previous actions. Specifically, children who had seen the experimenter pick up the object moments earlier, and thus knew the experimenter wanted the object, picked up the object and returned it to the experimenter. Meanwhile, children who had seen the experimenter leave the object on the ground did not pick up the fallen object, inferring the experiment’s desire to leave the object on the ground. At 22, 25, and 28 months of age, 33%, 50% and 75% of children, respectively, picked up the object at least once during the experimental condition. Critically, children were able to identify the goals of the experimenter and act accordingly. These findings provide the first experimental evidence that proactive helping behaviors may emerge in early childhood, at least in North America. This suggests that young children are not only able to detect the intentions of an individual, but also adjust their behavior to help satisfy the goals of the individual–a remarkable feat for a young child.

### The challenges of generalizing from North American samples

Do children outside of an urban, Western, middle-class society engage in proactive helping? No study has directly tested this question but a growing body of research suggests that it is problematic to assume phenomena observed within one society generalize to humans elsewhere. Indeed, Henrich and colleagues reviewed empirical studies in the behavioral sciences and report significant differences between Western, educated, industrialized, rich and democratic (WEIRD) societies (i.e. those who make up the majority of the participants in social science research) and rural, non-Western societies who comprise the majority of the human population [21]. Echoing this sentiment, Nielsen and Haun argue that an understanding of child development is incomplete without cross-cultural and comparative studies. In making this claim, the authors reviewed the developmental (and comparative) literature and report striking differences between cultural groups on a variety of domains in developmental science, such as theory of mind and social learning, as well as cooperation and prosocial development [22]. Critically, Nielsen and Haun describe culture-specific pathways in children’s prosocial behavior and reactions to unfairness. For instance, recent research demonstrates that although children from diverse cultures display similar prosocial tendencies early in life, these tendencies adjust to cultural norms as children reach adolescence [23]. Similarly, children’s reactions to unfairness follow differential developmental trajectories across seven diverse societies [24]. Together, these findings suggest that early social experiences may shape the developmental foundation that was once assumed to be similar across cultural groups. Indeed, cross-cultural and comparative studies can help determine which features of prosociality are sensitive to cultural variation and which features remain stable. The current project contributes to a growing body of work by examining whether young children from a rural, non-Western society in Vanuatu engage in proactive helping.

### Proactive helping & culture

Given research suggesting that it is problematic to assume phenomena observed within an urban, Western setting generalize to humans elsewhere, the current research builds on earlier work by examining whether the proclivity to engage in proactive helping is detectable in remote, small-scale, rural villages on Tanna Island in Vanuatu. On Tanna Island, individuals live a subsistence-based livelihood with no electricity or piped water and very little emphasis on formal education. A subsistence-based collective livelihood has been argued to produce a child rearing environment that is distinct from an urban, Western and independent lifestyle [25–30]. Closely examining child development in a drastically different environment, such as Tanna, offers insight into whether phenomena detected within North America and Europe are culture-bound or generalize beyond an urban, Western settings [31]. For example, is the propensity to infer the desires of another individual a trait shared by humans living in societies where mental states are likely less frequently discussed or used to guide behavior [32]? Or, will children develop the same motivation to help others in societies with different early child rearing practices than North American? In other words, is proactive helping sensitive to cultural variation in early social experiences?

There are at least two possible explanations for why proactive helping may differ across cultures. First, children’s responsibilities and societal expectations regarding their role as helpers vary across cultures. Decades of detailed ethnographies suggest that children’s domestic responsibilities vary across diverse socio-cultural contexts [26, 28, 30, 33–35]. Compared to urban, middle-class Western societies, children living in rural and also non-Western contexts are reported to be more responsible for childcare, food preparation, food gathering, and other household chores. This is supported by recent research by Köster and colleagues, who report higher levels of reactive helping among young children in rural Brazil as compared to children in urban Germany [6]. The authors argue that this difference may emerge from caregivers’ socialization goals and practices that vary as a function of the necessity of children’s domestic responsibilities to the survival of the family. Indeed, caregivers are more likely to emphasize obedience, conformity with social norms, and caring for others when these behaviors are functionally relevant, such as when children are required to care for a large number of siblings or provide subsistence of the family. Second, differences in children’s mental state understanding–the ability to represent another person’s intentions or desires–may lead to differences in children’s proactive helping behaviors. There is a growing body of evidence that suggests there is cross-cultural variability in the age at which children develop mental state understanding [36–41]. However, other evidence suggests mental state understanding may develop in a similar manner across diverse social contexts [42, 43].

Proactive and reactive helping are similar in many ways, as they both require an individual to recognize a need for help, be motivated to act, and act accordingly. However, these two forms of helping differ in interesting ways and may be driven by different motivations [44]. Proactive helping may be more demanding than reactive helping because it requires an actor to infer another person’s need from more subtle cues, such as the situation or previous actions rather than concurrent behavior or communicative cues. Therefore, although past research has found that young children across diverse cultures engage in reactive helping, variation has also been identified in the frequency with which children help and motivations underlying their prosocial behavior [5–7]. For example, Callaghan and colleagues found that two-year-olds in Canada reactively helped more than those in Peru and India [5]. Additionally, it is plausible that proactive helping is distinct from reactive helping and therefore may vary depending on the propensity for an individual (or a group) to factor in others’ perspectives. To the extent that proactive helping relies on some aspect of mental state understanding above and beyond reactive helping, we can predict that rates of proactive helping may vary across cultures. Although only a handful of studies investigating mental state understanding across cultures exist, they do suggest that there is significant cross-cultural variability in this complex ability [39].

#### Current research

In the present paper we provide the first direct test of whether children between the ages of two to five years from rural Vanuatu engage in proactive and reactive helping. Children from rural Vanuatu provide an interesting and important comparison to North American children because several key correlates of prosocial behavior may differ across cultures (e.g., domestic responsibilities and mental state understanding). Replicating the experimental protocol designed by Warneken [4], we examined whether young children in a rural, isolated non-Western society on Tanna Island, Vanuatu demonstrate a propensity to help in the absence of a clear request (*proactive helping)–*see Methods section. We also examined children’s behavior on a *reactive* helping test [2] expecting to replicate earlier findings in which children from non-Western urban environments help at above chance rates [5–7]. The current study extends previous research on helping across cultures in at least two ways. First, it examines a wide age range of children in a rural, isolated, small-scale society–one where knowledge of and exposure to Western norms is limited–providing a strong test of generalizability for findings initially demonstrated among young children in industrialized nations. Second, it examines proactive helping, a unique form of assistance wherein a child provides spontaneous help without a direct request. The purpose of the current study was to test whether proactive and reactive helping are detectable in this context. The relationship between helping and cultural dimensions such as mental state understanding and domestic responsibility is an empirical question for future research.

The detection of proactive helping in children from Vanuatu would provide evidence for cross-cultural consistency of this ability. Cross-cultural consistency of proactive helping would support a theoretical model suggesting that this ability may develop similarly across different social groups and therefore underscore its significance to human social life. Alternatively, if proactive helping behavior is absent in young children in a diverse social environment, it may suggest that this kind of prosocial behavior is sensitive to variations in environmental features, such as those described above (e.g., domestic responsibilities and mental state understanding). Given existing evidence of cross-cultural similarity in prosocial behaviors [5–7], coupled with evidence of emotional benefits of prosocial behavior [45], we hypothesize that children in Vanuatu will help both reactively and proactively similar to children in Western cultures. However, to the extent that children’s prosocial development is sensitive to cultural factors such as domestic responsibility or mental state understanding, children in Vanuatu may differ in their proclivity to help reactively and/or proactively.

## Methods

A total of nineteen children from rural Vanuatu between the ages of two to five years old (*X*_*age*_ = 45 months, *SD* = 9.6; 14 males) participated in this experiment. Five additional children were tested (*X*_*age*_ = 39.13, *SD* = 20.7; 3 males) but excluded from analyses due to fussiness (3) and experimenter error during the procedure (2). All children were recruited by word of mouth and birth records were used to determine age. We recruited all participants falling within this age range in the villages where the research was conducted. Due to the low population in the villages (50–100 people) there were no other children available to test during our visit. All data collection was approved by the Office of Research Ethics at Simon Fraser University and appropriate permits were obtained from the local Cultural Centre on the Island of Tanna, Vanuatu.

### Location & general procedure

We selected Tanna Island, Vanuatu as our testing site because the population differs from the North American sample examined by Warneken [4] in several ways, providing a strong test of generalizability. The children in this study were recruited from three nearby villages in central Tanna. Each of the villages contains fewer than 100 people, often living in smaller family units. People in rural Vanuatu rely on subsistence living, with most households producing their own food or selling to the local village market. Homes are small dwellings constructed out of local plant materials and villages do not have electricity or running water.

Early childhood experiences on Tanna differ in three important ways from that of an urban, Western context. First, Tanna is a subsistence island society. The result is that much of adult and child life is spent attending to crops, food gathering, harvesting and fishing. The food insecurities of this livelihood may also result in more responsibility on the child to partake in domestic chores. Second, there is little access to market economy and Westernization. This means that people rarely have opportunities to travel off of the island or gain access or knowledge of Western parenting ideals. Third, the people on Tanna (and Vanuatu in general) are thought to lead a collective or interdependent lifestyle, compared to an independent or individualistic lifestyle of many urban, Western societies. These differences in (1) responsibility of the child, (2) small-scale island life, and (3) inter-dependent lifestyle may require different social-cognitive skills than children growing in an urban, Western environment. For example, life on a small island society may not require individuals to rely on mental state understanding to succeed in that society. Individuals may rely primarily on observable behavior of others. Alternatively, it could be that children are precocious in their mental state understanding as well as their propensity to help due to the expectations on children to help and understand from a young age. As a result, early social life may differ in important ways with a differential emphasis on theory of mind and other social-cognitive elements.

On Tanna Island, the lead author trained a local female on the scripts and procedure for the study. This local female adult conducted the experiment in the child’s primary language (oral village dialect or Bislama, the official language of Vanuatu). Abbreviated versions of the scripts used by Warneken [4] were translated into Bislama and back-translated to English by another research assistant to ensure accuracy (see supporting information). The Bislama scripts were then translated to the local dialect in the village and once again translated to English to ensure accuracy. An independent research assistant, unaware of the study details, conducted each translation step. Discrepancies in meaning resulting from translation were discussed and corrected.

Children were recruited by word of mouth and tested in a quiet room in a vacant local school building with their caregiver. Prior to being brought into the testing room, caregiver consent and child assent were obtained by the local experimenter. Only the child and the local experimenters were visibly present in the room for testing, the parent remained outside while the lead author remained out-of-sight within the testing room. Upon completion of testing, all families were compensated individually with a small gift (toy/candy) and a donation was made to the village for hosting and facilitating the research project.

### Procedure

In order to provide consistent tests of children’s helping behavior, we used Warneken’s protocol to examine proactive helping [4] and we used Warneken and Tomasello’s protocol to examine reactive helping [2]. The procedure was divided into a warm-up and two testing sessions (assessing proactive and reactive helping, respectively). While proactive and reactive helping both provide children with the opportunity to assist someone in need, reactive helping presents helpers with a clear request for assistance. In contrast, proactive helping does not provide a direct request for assistance; helpers must recognize that a target is in need, even when the target themselves is unaware. Each child was randomly assigned to either an experimental (*n* = 10) or control (*n* = 9) condition (between subjects). All children participated in the proactive test first, followed by the reactive test (within subjects). We prioritized the proactive helping task given the lack of cross-cultural research on this specific behavior and strong potential for order effects if the reactive test were conducted first. The testing space was set-up similar to Warneken [4], with one room divided into three general areas: a free play, demonstration, and testing area. There was a table set up in both the demonstration and testing area, with cans and papers strewn about on the tabletop prior to the child entering the room.

#### Warm-up

The study began with a warm-up session in which the primary experimenter (E1) engaged in playful activities with the child (e.g. rolling a ball back and forth and locating hidden balls in the room) to ensure that the child felt comfortable moving about the testing location. Next, the child’s willingness to approach and interrupt E1 while she appeared busy was evaluated using a social initiation test. For this test, the child was instructed to interrupt E1 to show her a toy while she appeared busy. All children were willing to interrupt E1 to show her the toy and thus passed this test. Subsequently, a new toy was introduced to keep the child stationary and independently engaged while facing E1, enabling a clear view of the experimenter’s activities.

#### Proactive helping test

As in Warneken’s original research [4], the proactive test consisted of two identical blocks, each containing two phases: the exposure phase and the test phase. Each child saw two exposures trials during the exposure phase, followed by the test phase with 3 test trials for each block. The exposure phase was designed to manipulate the perceived intentions of E1 in the control versus the experimental condition. In both conditions, E1 demonstrated her desire to have the cans and papers on the exposure table put away into the empty basin. She announced, “Oh no! Look at that mess! I need to clean that up!” and proceeded to place cans and paper into the basin located on the demonstration table. After 15 seconds of tidying, E1 lifted a can from the demonstration table, surreptitiously causing another can to fall to the ground. As the can hit the floor it made an audible crash causing E1 to suddenly notice and look toward the fallen can. In the *experimental condition* she exclaimed, “Oh no, my can fell!” and picked the can up. Although the experimenter is not explicit in her intentions (i.e., does not verbally state that fallen cans should be picked up), her intentions can be inferred based on her behavior (i.e., she picked up the can). In the *control condition* she demonstrated indifference toward the fallen can by commenting, “I don’t need that one” and leaving the can on the floor. This demonstration was repeated once, to a total of two demonstrations. Each child had to witness at least one of the two demonstrations to be included in the analyses. If the child was looking in the direction of E1 during the exposure phase, or looked up immediately after the can fell, they were considered to have witnessed the event. All children met this criterion. This control condition allows us to conclude that kids do not rush to pick up any fallen object; they only do so when that is what someone wants. Additionally, this design holds constant the level of social interaction and play across conditions, making it unlikely that either of these explanations account for the observed tendency to inform the adult of the fallen can when required (i.e. provide proactive assistance), but not when unnecessary (i.e. in the control condition).

After observing the experimenter’s desires in the demonstration phase, children were presented with the test phase, which was identical for both conditions. The test phase consisted of three identical trials occurring in quick succession, providing three short opportunities (<5 seconds each) for the child to act. In each trial E1 continuously tidied the mess of papers and cans on the test table. While doing so, one can “accidentally” rolled off the table and landed within the child’s view. E1 continued cleaning without pause, suggesting that she was unaware that the can had fallen. Children’s responses were categorized as providing help or not–see Dependent Measures and Coding for detail. Regardless of children’s behavior during the study, the experimenter remained neutral. If the child picked up the can and handed it to the experimenter, she placed it on the table with a neutral expression and did not explicitly acknowledge the child.

#### Reactive helping test

Next, we assessed children’s propensity to engage in reactive helping, measured here by providing assistance to an adult experimenter following non-verbal expression of need [2]. After the proactive session ended, E1 returned to the free play area on the floor to play with child and the distracter toy. After one minute of play, E1 announced that she had to do some work and sat at the test table facing the child, who remained seated by the distracter toy. E1 began drawing on paper with a crayon. In both conditions, E1 “accidentally” dropped a crayon onto the floor and out of her reach. In the *experimental condition*, E1 reached unsuccessfully in the direction of the crayon, demonstrating her intention to retrieve the crayon. Importantly, E1’s gaze remained on the crayon while reaching for the object and she made a single verbalization of distress (i.e., “Ah!”), accompanied with a surprised/distressed facial expression. If the child did not retrieve the crayon after approximately five seconds, E1 oscillated her gaze between the child and the crayon. If the child still did not retrieve the crayon after an additional five seconds, E1 said “My crayon!” while gazing at the crayon. In the *control condition*, E1 did not reach for the crayon and instead placed her hands on the table, thereby demonstrating her intention to leave the crayon on the floor. E1’s gaze remained towards the child with a neutral facial expression. Only one test trial was conducted for the reactive session.

### Dependent measures & coding

Two independent coders (blind to condition) watched each edited video recording of the isolated and unidentifiable test trials during the proactive and reactive sessions to identify instances of helping. Both coders watched all videos. Children’s helping behaviors were coded as either instrumental helping or informing, and combined into an overall measure of helping for analyses. Using Warneken’s definitions [4], instrumental helping was defined as picking up the can and either handing it directly to the experimenter or placing it on the table. Informing was defined as any verbal or nonverbal attempt to alert the experimenter to the can (e.g., “Your can!” or pointing towards the can). Inter-rater reliability was high for instrumental helping (**κ** = .93). Informing was observed in only one child and was recognized by each independent coder.

To ensure that children witnessed the event, and were thus aware of the potential helping situation, we also coded children’s gaze behavior immediately following the can drop (**κ** = 0.83). Analyses are based on trials in which children actually witnessed the can-drop, indicated by them looking up at least once during the test phase [4], thus excluding one of the two test phases for four participants (two children from the control condition, two children from the experimental condition). All children attended to the fallen can at least once during the session.

## Results

### Proactive helping

**Fig 1:**
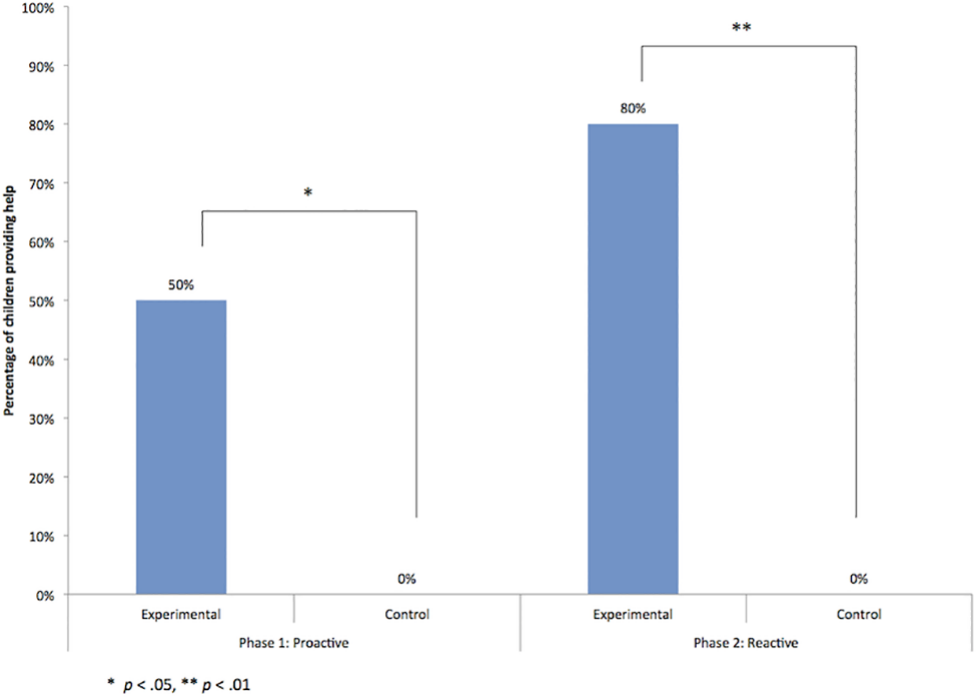
Percentage of participants who helped in the proactive and reactive helping tests by condition.

Five out of ten children in the experimental condition offered proactive help, yet none of the nine children in the control condition did (see Fig 1). A Fisher’s exact test revealed that children were significantly more likely to help in the experimental condition than the control, X^2^ (1, 19) = 6.107, *p* = .033, *r* = 0.57. Four children in the experimental condition provided instrumental help on at least one trial, by picking up the can and either placing it on one of the tables, or handing it directly to the experimenter. Two of these four children handed the can to the experimenter on five out of six trials; one child handed the can to the experimenter on three out of six trials, and one child handed the can to the experimenter on three trials and placed the can on the table on three out of six trials. One additional child did not help instrumentally, but informed the experimenter by pointing at the can while gazing towards the experimenter during one trial.

### Reactive helping

Children in the experimental condition were also significantly more likely to provide reactive help than children in the control condition. Eight out of ten children in the experimental condition provided reactive help, and again none of the nine children in the control condition did (Fig 1). There was a significant effect of condition as indicated by a Fisher’s exact test, X^2^ (1, 19) = 12.436, *p* = .001, *r* = 0.81. Of the eight children who provided reactive help in the experimental condition by retrieving the crayon from the floor, six children handed the crayon directly to the experimenter and two children placed it on one of the tables. All of the children who provided instrumental help in the proactive test also offered instrumental help in the reactive test; four additional children helped reactively who did not help proactively.

Despite random assignment, children in the experimental condition were marginally older (*X*_age_ = 48.2, *SD* = 9.67) than children in the control condition (*X*_age_ = 40.7, *SD* = 8.29), *t*(17) = -1.804, *p* = .089. However, age did not appear to account for the difference in helping across conditions as age was not significantly correlated with helping in the experimental condition on either the proactive, *r*(8) = -.38, *p* = .28, or reactive test, *r*(8) = .26, *p* = .47. No child of any age proactively or reactively helped in the control condition (*n* = 9). Thus, given that age was not predictive of helping behavior and no child helped in the control condition, it is unlikely that the difference in age is responsible for our findings. There was no difference in the age of children who helped reactively (*X*_age_ = 50.15 months, *SD* = 9.69), compared to children who helped both proactively and reactively (*X*_age_ = 48.15 months, *SD* = 9.77), *U* (*N* = 10) = 6, *Z* = -0.577, *p* = .564. Gender was not correlated with either proactive or reactive helping, *r*(19) = -.186, *p* = .45 and *r*(19) = -.217, *p* = .37, respectively.

## Discussion

Two- to- five-year-old children in rural Vanuatu provided help to others, even in the absence of overt behavioral cues from the person in need of help. Children were able to use prior knowledge to guide their behavior and act accordingly. In fact, only children in the experimental condition (where the experimenter previously demonstrated a desire for a fallen object to be picked up) provided help. Five out of ten children (50%) in the experimental condition helped at least once to remedy the unnoticed accident. In contrast, no child in the control condition (where the experimenter previously demonstrated a desire to leave a fallen object on the ground) provided help to the experimenter. Of the nine children in the control condition, the fallen object was not picked up a single time. In the reactive helping test, eight out of ten children (80%) helped an experimenter after she indicated her need for assistance with overt behavioral cues (e.g. reaching). In the control condition, when the experimenter did not produce these cues, none of the nine children provided help. The finding that more children engaged in reactive helping than proactive helping aligns with previous findings indicating that concurrent behavioral cues facilitate helping behaviors [10]. However, although such cues are facilitative, they are not always necessary for children to intervene. A possible explanation as to why concurrent behavioral cues are facilitative of helping behaviors is that they decrease the reliance on an understanding of the mental states of others.

Although these findings can only be discussed in terms of the specific cultural context studied, as is the case with any research conducted in a single context (e.g., Western contexts), the current study adds to an accumulating body of research providing insight into the development of helping behaviors in children worldwide. Young children from a cultural community that is distinct from those typically studied take action to intervene on another’s behalf, even in the absence of concurrent behavioral or communicative cues indicating that help is necessary. The absence of these cues occurring prior to the helping behavior is important, as there is no behavioral prompting that could be interpreted as a request for help. Although children are bystanders engaged in their own task, they stop what they are doing and act prosocially. Several theories might explain the finding that young children in this remote region of the world engage in helping behavior in ways that are similar to children in urban, Western societies. It is likely that children are socialized in similar *and* different ways to help others throughout their lives. Although alternative interpretations of young children’s helping exist [19, 20], they do not explain our finding that children in the experimental but not the control condition helped. For example, one theory of young children’s helping is that the behavior is motivated by empathic concern following the perception of another in need. However, given that proactive helping occurs in the absence of an emotional expression from the helpee, others have argued that empathetic concern is unlikely to be the driving factor [20]. Additionally, it may be argued that children’s helping behavior in the current research is imitative or rooted in observational learning. By watching the experimenter “pick up” the objects in the experimental condition, but not the control condition, children may be simply imitating this behavior. However, it is well-known that early helping behaviors reach far beyond imitative behavior with toddlers helping through pointing, sharing, instrumental actions (including novel ones) and combining these actions to help effectively. As such it is unlikely that children are helping solely as a result of imitative learning. Finally, recent research supports the interpretation of this behavior as rooted in prosocial motivations and concern for others’ well-being [46].

The purpose of this study was not to examine the complex interplay of early experience and development. Rather, our goal was to determine whether proactive and reactive helping behaviors are present in young children in a unique cultural context. This investigation and the resulting findings are particularly valuable in light of recent concerns explaining why it is problematic to assume that findings from children in any one context generalize to children elsewhere [21, 22]. The current findings lend support to a theoretical model suggesting that some aspects of prosocial development may emerge early in life despite vast cultural differences in early socio-cultural environment. Like children in urban, Western societies, children from Vanuatu attend to situational cues to guide their helping behavior. Although we did not test any precise mechanisms underlying why we find similarities in this behavior across these very different socio-cultural environments, we provide two possible explanations for these observed similarities below.

### Biological foundation hypothesis

Many would agree that growing up in a middle-class urban American society differs in various ways from growing up on a small-scale, rural, isolated island society in the South Pacific–with no electricity or plumbing. The differences are striking. What is even more striking, however, are the similarities detected in prosocial behavior across cultures. Although children and most parents on this island have only a few years of formal education and little-to-no exposure to Western media, willingness to provide proactive and reactive assistance appears to be quite similar. We find support for this hypothesis with the current study as children in Vanuatu factor in the behavioral cues and situational cues to guide their behavior in ways that are similar to what has been observed in American children. However, the current study examined helping behaviors in children of a broader age range than previously studied.

### Similar experience hypothesis

The majority of ethnographies produced over the last century indicate that early childhood experiences vary greatly across different societies [28]. However, it is possible that children are, in fact, exposed to similar experiences yet those experiences may manifest differently. For instance, children from all cultures may learn to negotiate and become prosocial by interacting with other children and caregivers, even if the specific socialization goals, situations, and practices differ across societies. Examining seemingly universal mechanisms across diverse social contexts is essential for understanding the subtle nature of development. Future research may provide insight into the relationship between children’s developing prosocial tendencies and early social experiences through direct measurement of these behaviors. For example, research regarding the scaffolding of young children’s early helping behavior suggests that socialization may play an important role in the development and emergence of children’s helping [16]. Further exploration and direct measurement of the ways in which children’s daily experiences may shape helping behavior would be beneficial to understanding the development of this complex ability [6, 7].

### Limitations

Despite its strengths, the current research is limited by a small sample size and wide age range of participants. Consequently, these results should be interpreted with caution. Nonetheless, these data are the first to examine proactive helping in a vastly different cultural context with an inclusive sample of young children from remote villages, and thus offer important insight. We tested all available children in these villages during our visit, and despite the small sample size (*n* = 19) we were able to detect a difference between the control and experimental conditions. To more fully understand the complex nature of this developing ability requires follow-up and multi-method procedures.

Previous studies of children’s reactive and proactive helping have generally focused on the second year of life, the current research included children from two to five years of age. The wider age range allowed here was necessary to reach a useable sample size. Most villages have only approximately 100 members with few children, let alone toddlers near the age of two. Young children in Vanuatu helped both reactively and proactively in the experimental condition despite the fact that the participants were from a wider age range than those previously tested. Although it may be argued that children’s motivations for helping may shift across development, the current research is the first to demonstrate that children’s reactive and proactive helping behaviors persist into early childhood. Additionally, although children were randomly assigned to the experimental and control condition, we did not measure abilities theoretically related to prosocial behavior (e.g., mental state understanding). We recognize that random assignment is less effective in smaller sample sizes, and therefore cannot discount the possibly of systematic differences in children’s mental state understanding or other cognitive abilities impacting our results.

A further limitation of the present work is that task order (reactive and proactive helping) was not counterbalanced across participants. This decision was intentional given our specific focus on proactive helping and, as such, the proactive helping task was presented first so children’s reactions would not be biased by prior explicit requests for help. Of course, this raises concern that rates of reactive helping could have been tainted by the proactive helping task, however, given that findings align with past research [5], it is unlikely the results are strictly a result of order effects. Additionally, past research has revealed that social interaction with the experimenter before a helping task can boost children’s helping behavior [47, 48]. Because testing phases in the present study always began with proactive helping followed by reactive helping, it is possible that children helped more in the reactive testing phase simply because they had more opportunity for free play with the experimenter prior to the reactive helping test. Critically, however, the additional interaction and playtime introduced between the proactive and reactive helping phases was consistent for both the experimental and control condition. As such, the additional interaction and playtime between testing phases cannot account for condition differences in the rates of helping behavior. Further, it is striking that no child in the control condition provided help in either the proactive or reactive helping task. However, this is congruent with past research on helping behavior where children usually demonstrate near floor effects in similar helping tasks [2, 4, 49]. Despite the similarity to results observed in industrialized or large-scale societies, it is also possible that children in Vanuatu may have been unlikely to respond to the experimenter in the control condition due to culture-specific factors. For example, ethnographic reports indicate that in many subsistence-based societies, obedient behavior is a highly emphasized socialization goal [50–52]. Therefore, it is possible that more emphasis on obedience among children in Vanuatu may have reduced the likelihood of intervening in the control condition.

Finally, the motivation underlying the observed prosocial behavior in the experimental condition, but not the control condition, is difficult to identify from this study alone, given that the current design does not completely rule out motivations beyond prosocial motivations (see Paulus for a review of the other interpretations and debate [20]). Children may have been motivated by 1) goal contagion, with young children adopting the goals of others and attempting to complete another’s goal as if it was their own [53], 2) a desire for social affiliation [19, 54], or 3) a learned behavioural response unrelated to prosocial motivation [20]. These are possible explanations for our current study, but based on the body of existing that has addressed these alternative interpretations [46, 55, 56], we feel that the prosocial interpretation is likely.

### Conclusion

The current research contributes to a growing body of knowledge on human prosociality and the development of human social cognition. Specifically, the current data suggest that proactive and reactive helping can be detected in a small-scale rural society outside of an urban, Western society. Although this behavior may be interpreted in a variety of different ways, these results support a theoretical model suggesting that prosocial tendencies exist across different social groups, despite vast cultural differences in the early socio-cultural environment. However, many questions regarding the development of diverse helping behaviors remain. Broadly, is helping a universal feature of early childhood necessary for human survival or is it a culture-specific adaptation that develops through unique pathways depending on the cultural context? Future research may help us better understand the development of prosocial behaviors, such as helping, by directly comparing different societies with direct predictions regarding how helping may vary with contextual factors.

Using a simple and transportable procedure, we examined whether proactive and reactive helping were detectable in a small-scale rural environment using procedures comparable to those used in North America [4]. To further examine the complexity of the behavior, as well as to fully appreciate the similarities and differences in the developmental pathways across cultures [53], we suggest a more comprehensive research program investigating the early social ecology in combination with experimental methods, detailed ethnography, and natural observations of everyday life [54, 57].

## Supporting information

S1 File Verbal script for reactive and proactive helping in English and Bislama. (SAV)
